# Serum Tumor Markers in Paraneoplastic Neurologic Syndromes: A Systematic Review of Guidelines

**DOI:** 10.3389/fneur.2020.607553

**Published:** 2021-01-18

**Authors:** Chiara Trevisiol, Ilaria Cani, Aline S. C. Fabricio, Massimo Gion, Bruno Giometto, Patrizia De Massis

**Affiliations:** ^1^Veneto Institute of Oncology IOV–Istituto di Ricovero e Cura a Carattere Scientifico (IRCCS), Padua, Italy; ^2^Department of Biomedical and NeuroMotor Sciences (DIBINEM), Alma Mater Studiorum–University of Bologna, Bologna, Italy; ^3^Regional Center for Biomarkers, Department of Clinical Pathology and Transfusion Medicine, Venice, Italy; ^4^U.O. Neurologia, Santa Chiara Hospital, Azienda Provinciale per i Servizi Sanitari (APSS), Trento, Italy; ^5^U.O. Neurologia, Azienda ULS Imola, Imola, Italy

**Keywords:** circulating tumor markers, paraneoplastic neurologic syndromes, practice guidelines, cancer diagnosis, quality of health care

## Abstract

**Purpose:** Algorithms for the detection of a malignancy in patients with unclear neurologic symptoms of suspicious paraneoplastic origins are not universally applied. Frequently, circulating tumor markers (TMs) are considered a valuable tool for cancer diagnosis in patients with paraneoplastic neurologic syndromes (PNS). Our aim was to extract the recommendations on the use of TMs and onconeural antibodies (Abs) for the diagnosis of malignancies in PNS from clinical practice guidelines and put them forward as evidence in a common framework to facilitate diffusion, dissemination, and implementation.

**Methods:** Systematic literature searches were performed for guidelines on both oncology and PNS published since 2007. Guidelines containing information and recommendations for clinical practice pertaining to the screening and diagnosis of PNS were selected. Information on circulating TMs and onconeural Abs was extracted and synthesized in consecutive steps of increasing simplification.

**Results:** We retrieved 799 eligible guidelines on oncology for the potential presence of information on PNS but only six covered treated diagnosis or the screening of cancer in PNS, which were then selected. Seventy-nine potentially relevant guidelines on PNS were identified as eligible and 15 were selected. Synoptic tables were prepared showing that classical TMs are not recommended for the screening or the diagnosis of a malignancy in patients with a suspected PNS. Neither should onconeural Abs be considered to screen for the presence of a malignancy, although they could be helpful to define the probability of the paraneoplastic origin of a neurologic disorder.

**Conclusion:** The present work of synthesis may be a useful tool in the diffusion, dissemination, and implementation of guideline recommendations, potentially facilitating the decrease of the inappropriate use of circulating biomarkers for cancer screening in the presence of PNS.

## Introduction

Paraneoplastic neurologic syndromes (PNS) are neurologic immune-mediated disorders occurring as a remote effect of a tumor, frequently associated with antibodies (1, 2). Antibodies (Abs) associated with PNS can recognize intracellular antigens (onconeural Abs) or bind to cell surface antigens on neuronal cells (3). While neuronal surface Abs often occur in the absence of tumors in non-paraneoplastic autoimmune diseases such as autoimmune encephalitis, onconeural Abs are closely related to the presence of a malignancy (4).

Neurologic symptoms suggestive of a PNS necessitate the proper diagnosis of PNS and the identification of a malignancy. Interestingly, evidence shows that PNS may occur in most cases before the clinical appearance of the malignancy, thus increasing the need for tools for early cancer detection in these patients (5, 6).

Laboratory tests are expected to play a key role in this diagnostic workflow due to the availability of screening tests for both antibodies and classical circulating tumor markers (TMs), e.g., CEA or PSA.

The presence of onconeural Abs increases the probability of a paraneoplastic neurologic syndrome (7) and several studies support clinicians on the appropriate interpretation of antibody tests results. Conversely, the detection of an underlying malignancy is more challenging, as the presence of antibodies is associated with risk rate, which is hardly applicable for diagnostic purposes for an individual patient (8).

Circulating TMs have an established clinical role to detect the relapse of malignancies during follow-up and to monitor the response to therapy in advanced disease, whereas they are not recommended for the early diagnosis of cancer due to their low sensitivity and specificity (9–11). Nonetheless, circulating TMs are widely requested for diagnostic purposes in clinical practice (12–18), being also ordered for patients with unclear neurological symptoms. Clinical practice guidelines are designed, disseminated, and implemented to help clinicians make appropriate diagnostic and therapeutic decisions. Thus, available guidelines should provide proper recommendations on the use of TMs and onconeural Abs for the diagnosis of occult malignances in PNS. However, the provided recommendations may not cover all clinical questions or may not be consistent throughout the guidelines produced in different contexts (19). Moreover, physician adherence is critical for translating guideline recommendations into improved healthcare, but a variety of barriers can affect the physician knowledge of guideline recommendations, undermining this process. These barriers include a lack of awareness and familiarity, caused by a high volume of information, scarce guideline accessibility, and the amount of time needed to stay informed (20).

Therefore, although numerous guidelines on PNS exist, this may not be sufficient to ensure evidence-based decision making. In fact, it was shown that the uptake of knowledge does not occur with simple dissemination (21), mainly in cases such as that of the use of TMs in PNS, in which a specific issue may be difficult to individuate in the body of comprehensive but multifaceted guidelines. The aim of this study was to apply a previously validated method (9) to systematically and critically review guidelines, extracting, synthesizing, and comparing, in a synoptic manner, the recommendations on the use of TMs and onconeural Abs for the early diagnosis of malignancies in PNS. The ultimate aim of this study was to document only recommendations on circulating biomarkers by structuring them in a common framework to facilitate their diffusion, dissemination, and implementation.

## Materials and Methods

Following a protocol designed by the authors and adherent to the Preferred Reporting Items for Systematic Reviews and Meta-Analyses (PRISMA) guidelines (22), two different independent systematic reviews of guidelines were sequentially undertaken to seek out recommendations or information on the detection of a tumor in patients with PNS. One review was focused on oncology guidelines and derived from a previous project, that lasted 5 years which had the objective of collecting and synthesizing recommendations from guidelines related to 18 different neoplasms [hereinafter referred to as the “Guide project” (9)]. The second review was focused on PNS guidelines.

### Search Strategy for Guidelines on Oncology

In the first phase of the study, 1,181 guidelines published from January 2009 to July 2015 were selected from the 8,266 documents retrieved from the bibliographic databases generated through the Guide project (9). The 1,181 full-texts were screened for the presence of recommendations and information for clinical practice related to PNS. The details for the guidelines selection have been previously published (9). In brief, a systematic search for guidelines was performed in bibliographic databases (PubMed, National Guidelines Clearinghouse, and GIN library). This first broader systematic search included key terms, their synonyms, and associated MeSH terms related to cancer and guidelines (see Pubmed strategy in Supplementary Table 1, Section A). Moreover, guidelines were searched in websites of organizations and scientific societies producing guidelines (Supplementary Table 2).

Documents containing recommendations for clinical practice were included. Reviews, technology assessments, commentaries to guidelines, and guidelines limited to sarcomas, hematological malignancies, pediatric population, and pregnant women, were excluded.

A standardized set of selection criteria was used to identify eligible publications addressing PNS (details are reported in Table 1).

**Table 1:** Eligibility criteria.

**Item**	**Inclusion criteria**	**Exclusion criteria**
Disease	Paraneoplastic limbic encephalitis; Brainstem encephalitis; NMDAR-Ab encephalitis; Paraneoplastic encephalomyelitis; Paraneoplastic cerebellar degeneration; Cerebellar ataxias; Subacute sensory neuropathy; Subacute motor neuronopathy; Subacute autonomic neuropathy; Chronic pseudo-obstruction; Paraneoplastic retinopathy; Neuromyelitis optica; Paraneoplastic opsoclonus-myoclonus; Paraneoplastic peripheral nerve hyperexcitability; Stiff-person syndrome; Progressive encephalomyelitis with rigidity and myoclonus; Acquired neuromyotonia; Morvan's syndrome; Lambert-Eaton myasthenic syndrome; Dermatomyositis; Myelitis	Chronic fatigue syndrome; Myalgic encephalomyelitis; Acute disseminated encephalomyelitis; Acute transverse myelitis; Primary autoimmune cerebellar ataxia; Gluten ataxia; Hashimoto's encephalopathy; Ataxia associated with systemic lupus erythematosus; GAD antibody associated cerebellar ataxia; Paraproteinemic neuropathies; CANOMAD syndrome; IgM paraproteinemic neuropathy; Myasthenia gravis; Guillain-Barrè syndrome; Miller Fisher syndrome
Clinical scenario	Screening; Diagnosis	
Language	English	
Type of publication	Guidance documents (documents containing recommendations and produced or endorsed by a government agency or professional health society)	Reviews, technology assessments, commentaries to guidelines, and service documents

### Search Strategy for Guidelines on PNS

A subsequent narrower search with MeSH terms and more specific keywords was conducted for identifying existing guidelines on PNS. Bibliographic databases were consulted including PubMed, National Guideline Clearinghouse, and websites of organizations and scientific societies producing guidelines (Supplementary Table 2). The full PubMed search strategy is shown in Supplementary Table 1, Section B. Any guideline containing information and recommendations for clinical practice related to PNS were eligible for inclusion (details in Table 1). Documents were included if they were published or updated between 2007 and 2017. A search for updated versions of the identified guidance documents was performed in January 2019 and the most up-to-date documents were included in the study.

Two authors (CT and IC) independently reviewed the two literature search results using the predefined eligibility criteria. Conflicts were resolved by discussion until a consensus was reached.

### Synthesis and Presentation of Recommendations

In the present study, we applied a novel approach to summarize and compare recommendations and other information on TMs published in guidelines that had been recently developed (9). During this previous project (9), guidelines on solid tumors were identified through systematic search, and their quality appraised; information on TMs were then extracted and summarized using the method summed up below, which has been designed to be explicit, verifiable, and reproducible.

The information related to the following clinical question was then searched in the selected guidelines: “In a patient with a suspected PNS, are circulating TMs and/or circulating antibodies recommended for the screening or diagnosis of any possible associated malignancy and its site of origin?”

The clinical information on diagnostic laboratory tests was searched, and data on circulating TMs and/or antibodies were extracted from every guideline and synthesized in a multistep process according to the previously published method (9). For each guideline, recommendations, implicit advices for clinical practice (not recognizable as explicit recommendations), and additional information concerning; (i) circulating TMs, (ii) antibodies, and (iii) the most commonly associated malignancies to a given syndrome were verbatim transferred in an (Excel) electronic sheet by one author (IC). Information extracted from the different guidelines was grouped with reference to the type of syndrome, according to the classification into “classical,” “non-classical,” and “others” subtypes (7). All the relevant information was summarized by one author (MG) and clustered in a single entry when different guidelines provided similar messages. The tabulated results were then appraised by three other authors (CT, IC, and PD) with methodological skills or specific clinical expertise on PNS. Every effort was made to avoid any interpretation of the content of guidelines and verbatim reporting of the original sentences were used whenever possible throughout the synthesis and clustering process. Comments and suggestions were discussed and resolved by consensus.

## Results

### Guidelines Focused on Oncology

**Figure 1:**
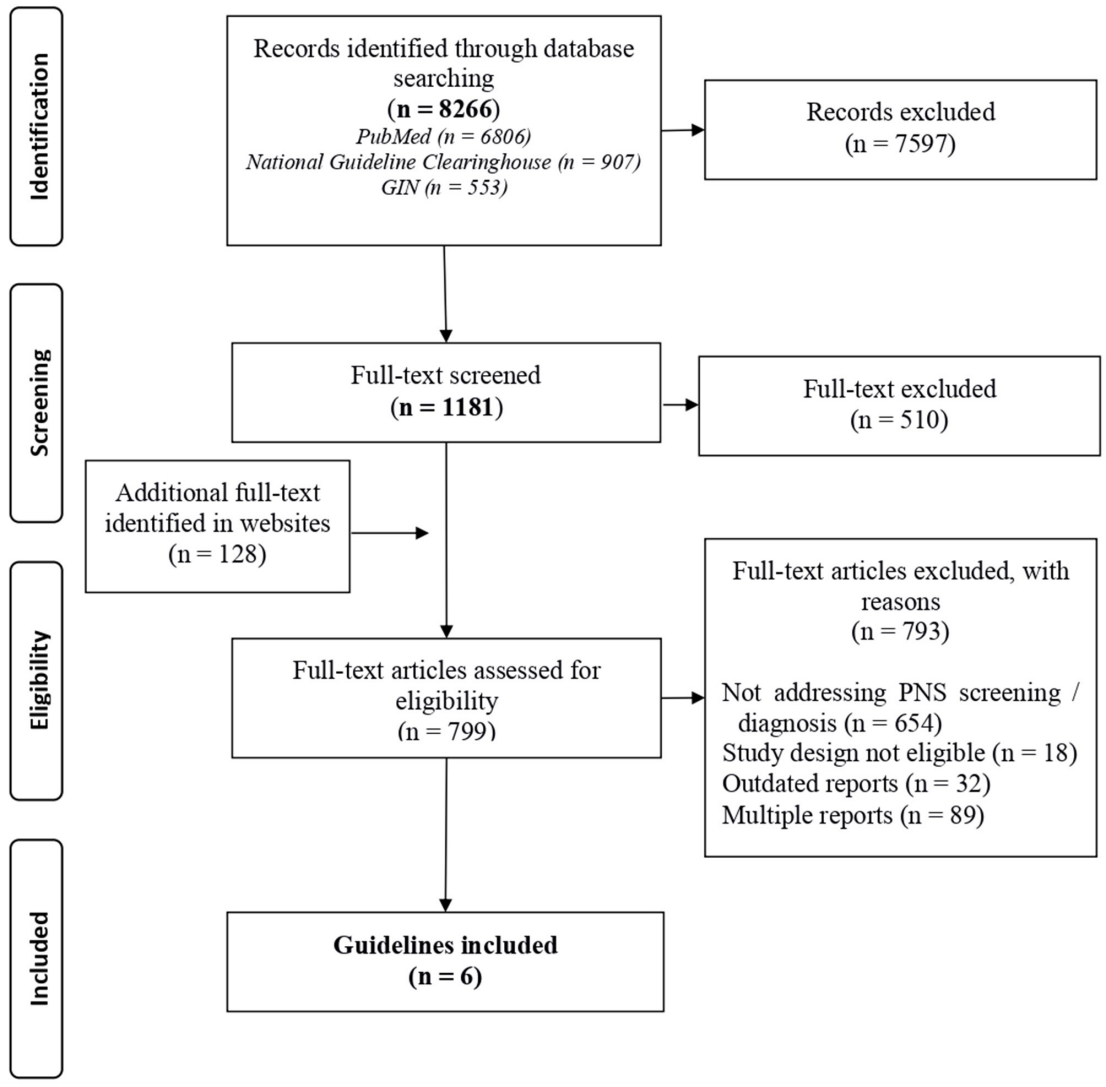
PRISMA 2009 flow diagram (modified) for guidelines focused on oncology.

Of the identified 1,181 guidelines focused in oncology in the Guide project, 671 were selected as they were eligible and contained the potential presence of information on PNS. In addition, 128 documents were identified in the website search. Of these 799 guidelines only 6 treated diagnosis or the screening of PNS and were selected (details in Figure 1, Supplementary Table 3). Three documents concerned lung cancer (23–25), one renal cell carcinoma (26), one pulmonary neuroendocrine tumor (27), and 1 was dedicated to screening for tumors in patients with paraneoplastic syndromes (28). Details on the recommendations and supplementary information on circulating TMs or antibodies for the screening or diagnosis of any possible concomitant malignancy are reported in Table 2. In summary, the guideline on neuroendocrine tumors supports the determination of 5-hydroxy-indole-acetic acid in 24-h urine and circulating adrenocorticotropic hormone (ACTH) and growth hormone-releasing hormone (GHRH) in the diagnostic work-up of the tumor, irrespectively, of the presence of PNS (27); two guidelines considered a diagnostic workflow in PNS cases but did not mention TMs (24, 26) one guideline considered the diagnostic workflow and mentioned TMs to underline their low diagnostic sensitivity and specificity (28); one guideline recommended against the use of TMs as a diagnostic tool (25); and one guideline reported the association of anti-Hu onconeural Abs in small cell lung cancer (SCLC), but did not provide clinical practice recommendations and did not address TMs (23).

**Table 2:** Summary of recommendations and supplementary information concerning circulating TMs and/or antibodies for the screening or diagnosis of the malignancies in patients with PNS from guidelines focused on oncology.

**Guideline acronym (ref.)**	**Malignancy(ies) addressed by the guideline**	**Summary of recommendations on TMs and/or circulating Abs**	**Additional information**
ACCC 2012 (26)	Renal cell carcinoma	Screening and/or diagnosis of cancer considered, but TMs and/or Abs not addressed	Paraneoplastic symptoms are present mainly in patients with very advanced disease, for whom the goal of treatment is palliation. It is therefore useful to perform additional diagnostic assessment for paraneoplastic symptoms, particularly if symptoms suggest paraneoplastic involvement
ACCP 2013 (23)	Lung cancer	Screening and/or diagnosis of cancer considered, but TMs and/or Abs not addressed	The initial evaluation of patients with lung cancer should include a thorough history and physical examination, pulmonary function tests, CT imaging, basic laboratory tests, and selective testing for distant metastases and paraneoplastic syndromes (most PNS in adults are associated with lung cancer of neuroendocrine origin). Anti-Hu syndrome is the PNS most relevant to lung cancer (anti-Hu antibodies have a sensitivity of 82% and a specificity of 99% in the diagnosis of PNS)
CCO 2014 (24)	Lung cancer	Screening and/or diagnosis of cancer considered, but TMs and/or Abs not addressed	A person should have a chest x-ray within two working days if they present with any of the following features suggestive of paraneoplastic syndrome
DGP-DKG 2011 (25)	Lung cancer	Screening for lung cancer with serum tumor markers is not recommended	
EFNS 2011-t (28)	Small cell lung cancer, thymoma, breast cancer, teratoma, ovarian carcinoma, testicular tumors, and other	Screening and/or diagnosis of cancer considered, no explicit recommendations on TMs, and/or Abs provided	Positive TMs raise the suspicion of a tumor, but normal values do not exclude malignancy as sensitivity is low to moderate (good practice point). Neuron-specific enolase (NSE), Progastrin-releasing peptide (ProGRP), CA-125, CA19-9, Alpha-fetoprotein (AFP), Carcinoembryonic antigen (CEA), and the beta-subunit of the Human chorionic gonadotropin (b-HCG) are mentioned with reference to different malignancies. Paraneoplastic antibodies are related to different PNS. The individual antibodies are referred to, but they are not described in detail. Other antibodies are not clinically related to specific PNS, but they have been described as specific biomarkers
ENETS 2015 (27)	Pulmonary neuroendocrine (carcinoid) tumors	Screening and/or diagnosis of cancer considered, but TMs and/or Abs not addressed	Paraneoplastic syndrome might occur in the setting of pulmonary carcinoids. Biochemical testing should be carried out in consideration of clinical symptoms and features including as appropriate 24-h urine 5-Hydroxy-indole-acetic acid, adrenocorticotropic hormone (ACTH), and growth hormone-releasing hormone (GHRH)

Clinical question is as follows: in a patient with clinical signs pertaining to a PNS, are circulating TMs and/or circulating antibodies recommended for the screening or diagnosis of any possible concomitant malignancy and its site of origin?

*TMs, tumor markers; Abs, antibodies; PNS, paraneoplastic neurologic syndrome*.

### Guidelines Focused on PNS

**Figure 2:**
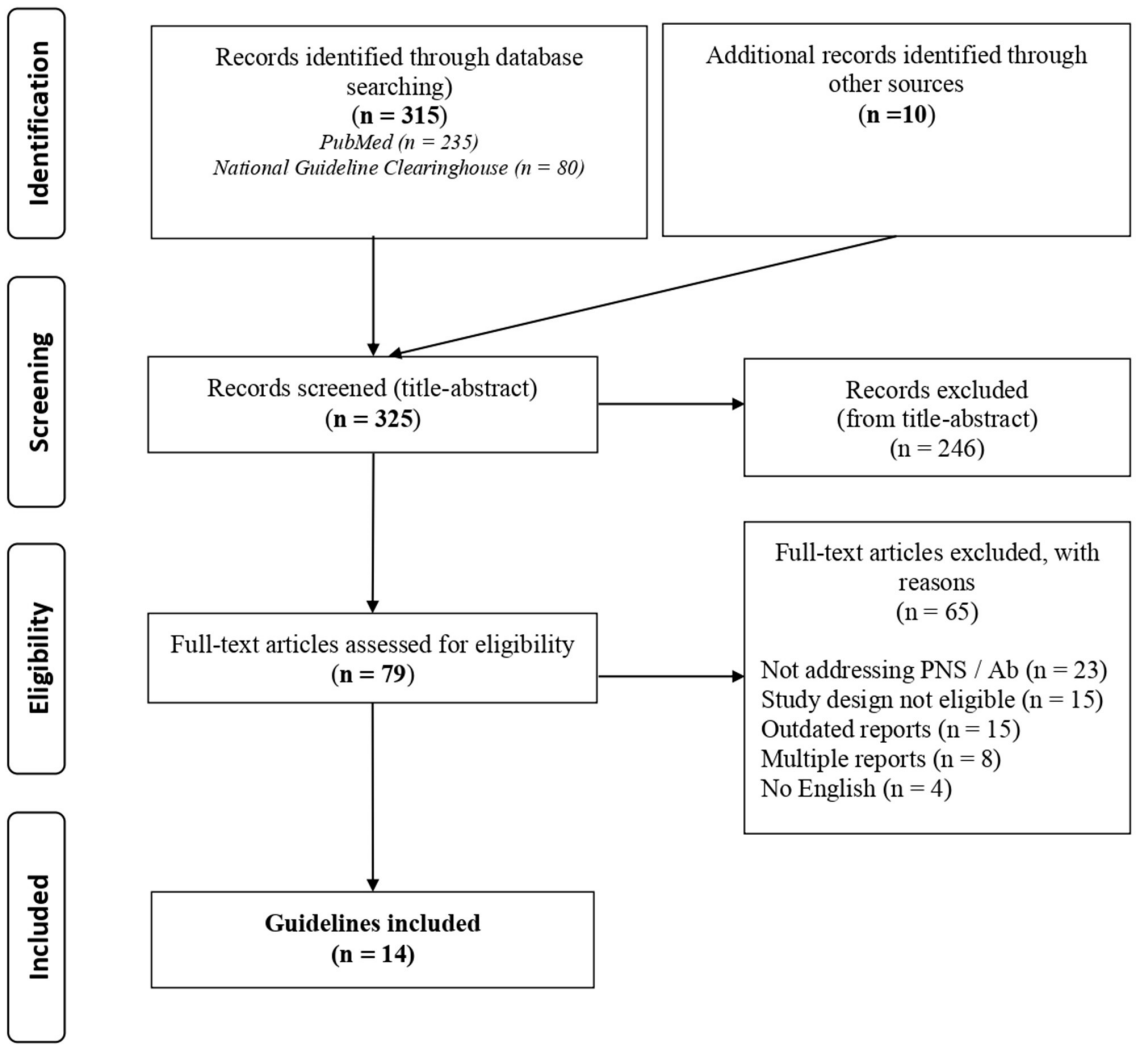
PRISMA 2009 flow diagram (modified) for guidelines focused on PNS.

A total of 325 records were identified with the search strategy regarding PNS; from these, 79 were selected by title and abstract and 14 were included on the basis of the assessment of the full text (details in Figure 2, Supplementary Table 4). Data concerning circulating antibodies and/or TMs suggested for the diagnosis of the malignancies and the corresponding summary of recommendations are reported in detail in Table 3. The recommended strategies for the detection of possibly associated tumors in each PNS type are summarized in Table 4. Additional information regarding antibodies recommended to characterize each syndrome and the most commonly associated malignancies are reported in Supplementary Table 5.

**Table 3:** Summary of recommendations and supplementary information concerning circulating TMs and/or antibodies for the screening or diagnosis of the malignancies in patients with PNS from guidelines focused on PNS.

**Suspected syndrome**	**Circulating TMs and Abs reported to be associated to an increased risk of malignancy*^a^^,^^b^***	**Malignancies reported to be associated to the PNS**	**Summary of recommendations and supplementary information concerning circulating TMs and/or Abs for the screening or diagnosis of the malignancy^**c**^**	**Guidelines that consider the specific PNS (ref)**
**CLASSICAL CENTRAL NERVOUS SYSTEM**				
Limbic encephalitis (LE)	Anti-Hu (SCLC), anti-Ma2 (testicular cancer), anti-CV2/CRMP5 (thymoma, SCLC), *anti-VGKC* (thymoma, SCLC), *anti-GABAb-R* (SCLC, thyroid, breast cancer), *anti mGluR5* (Hodgkin's lymphoma) *anti-AMPA-R* (SCLC, breast, thymoma) Circulating TM: NR	SCLC, testicular cancer (germ cell tumors), breast cancer, ovarian teratoma, Hodgkin's lymphoma, thymoma	Patients older than 40 years, smokers, and with the Hu antibody have to be investigated for the presence of a SCLC. Anti-Hu positive patients could also have extra thoracic tumors (EFNS 2011-pns) In male patients younger than 40 years, the detection of Ma2 antibodies suggests the presence of testicular cancer (EFNS 2011-pns) CV2/CRMP5 antibodies are instead detected in patients with thymoma or SCLC (EFNS 2011-pns) VGKC antibodies suggest the diagnosis of idiopathic limbic encephalitis, but in few cases VGKC can be associated with paraneoplastic LE and thymoma and SCLC (EFNS 2011-pns, PNSE 2004) GABAb-R are probably the most common antibodies found in LE in association with SCLC (EFNS 2012-cns)	EFSN 2011-pns (29) EFNS 2012-cns (4) EFNS 2012-d (30) PNSE 2004 (7)
Autoimmune encephalitis (AE)	*Anti-NMDA-R* (ovarian teratoma, rarely SCLC), *anti-VGKC complex* [Morvan's syndrome] (thymoma) Circulating TM: NR	Ovarian teratoma, thymoma	A minority of cases of AE (depending on surface or receptor antibody reactivity) are paraneoplastic forms (AINI 2017) Up to 50% of young adult female patients with anti-NMDA-R encephalitis have an ovarian teratoma or more rarely SCLC, this is much less common in children (EFNS 2012-cns) Around 40% of patients with Morvan's syndrome have tumors, often recurrent or malignant thymomas (EFNS 2012-cns)	AINI 2017 (31) EFNS 2012-cns (4)
Opsoclonus–myoclonus (OMS)	NR	*Adults:* SCLC, breast cancer, gynecological cancer (ovary, uterus) *Infants:* neuroblastoma	A paraneoplastic form of OMS is more common in older women and associated with breast cancer and SCLC (EFNS 2012-cns) In infants, the most common associated tumor is neuroblastoma (EFNS 2011-pns, EFNS 2012-cns)	ACCP 2013 (23) EFSN 2011-pns (29) EFNS 2012-cns (4)
Subacute paraneoplastic cerebellar degeneration (PCD)	Anti-Yo (ovarian, breast cancer), anti-CV2/CRMP5 (SCLC), anti-Hu (SCLC), anti-Ri (breast, lung cancer), anti-Tr (Hodgkin's disease), anti-VGCC (lung tumors), anti-mGluR1 (Hodgkin's disease) Circulating TM: NR	Ovarian cancer, breast cancer, SCLC, Hodgkin's lymphoma	The anti-Yo syndrome is commonly associated with ovarian and breast cancers (ACCP 2013, EFNS 2011-pns) The most frequently associated tumor to anti-CV2/CRMP5 is SCLC (60%) (EFNS 2011-pns) Patients with anti-Hu differ from those with anti-Yo in terms of a frequent association with SCLC (EFNS 2011-pns) The associated cancers to anti-Ri are breast or lung cancer (EFNS 2011-pns) Anti-Tr are markers of Hodgkin's disease (EFNS 2011-pns) Anti-VGCC (P/Q type) were demonstrated to be present in some cases of cerebellar degeneration in association with lung tumors (EFNS 2012-cns, PNSE 2004)	ACCP 2013 (23) EFSN 2011-pns (29) EFNS 2012-cns (4) PNSE 2004 (7)
**CLASSICAL PERIPHERAL NERVOUS SYSTEM**				
Subacute sensory neuronopathy (SSN)	Anti-Hu (cancer), anti-CV2/CRMP5 (SCLC, neuroendocrine tumors, thymoma) Circulating TM: NR	SCLC 70–80%, breast cancer, ovarian cancer, sarcoma, Hodgkin's lymphoma	The estimated specificity of anti-Hu in the diagnosis of cancer in patients suspected to have SSN is 99% and sensitivity is 82% (EFNS 2011-pns) SCLC, neuroendocrine tumors, and thymoma are usually associated with anti-CV2/CRMP5 (EFNS 2011-pns)	EFSN 2011-pns (29)
**CLASSICAL NEUROMUSCULAR JUNCTION AND MUSCLE**				
Lambert-Eaton myasthenic syndrome (LEMS)	Anti-VGCC (SCLC), anti-SOX (SCLC) Circulating TM: NR	SCLC >90% rarely reported: NSCLC, prostate carcinoma, breast carcinoma, thymoma, lymphoma	Nearly 50–60% of patients with LEMS have an underlying tumor, usually SCLC (EFNS 2011-pns, GISMIA 2014) Anti-VGCC are present in the serum of at least 85% of the patients of LEMS with or without SCLC (EFNS 2011-pns) Seronegative LEMS has a lower association with SCLC (GISMIA 2014) Factors associated with a higher risk of SCLC are older age (<50 years), rapid disease progression, high anti-VGCC antibody levels, and smoking (GISMIA 2014) The SOX antibody is present in 64% of the patients with LEMS and SCLC, and in 22% of the patients with SCLC and no clinical paraneoplastic syndrome. The antibody is absent in about one-third of LEMS patients with a SCLC (EFNS 2011-pns, GISMIA 2014)	ACCP 2013 (23) EFSN 2011-pns (29) GISMIA 2014 (32)
Dermatomyositis (DM)	NR	Breast cancer, ovarian cancer, gastrointestinal cancers (pancreatic, gastric, colorectal), lung cancer, Non-Hodgkin's lymphomas, nasopharynx carcinoma (in Asian descent)	~30% of all DM cases are tumor-associated (AWMF 2016)	ACCP 2013 (23) AWMF 2016 (33) EFNS 2011-t (28)
**NON-CLASSICAL, PERIPHERAL NERVOUS SYSTEM, AND CENTRAL NERVOUS SYSTEM**				
Peripheral nerve hyperexcitability (PNH) syndromes (Stiff person, neuromyotonia, progressive encephalomyelitis with rigidity and myoclonus-PERM)	Anti-amphiphysin (breast cancer, SCLC), anti-Hu (SCLC), anti-VGKC (thymoma) Circulating TM: NR	Thymoma, SCLC, NSCLC, Hodgkin's disease	Anti-VGKC are found in 80% of patients with thymoma (anti-VGKC antibodies are found also in about 35% non-paraneoplastic PNH) (EFNS 2011-pns) Hu antibodies can be helpful in PNH patients who had SCLC (EFNS 2011-pns) In PERM, one case of thymoma was reported (EFNS 2012-cns)	ACCP 2013 (23) EFNS 2011-pns (29) EFNS 2012-cns (4) PNSE 2004 (7)
**OTHER**				
Neuromyelitis optica spectrum disorders (NMOSD)	NR	Breast adenocarcinoma	Breast adenocarcinoma is the most frequent cancer associated with rare paraneoplastic NMOSD (AINI 2017)	AAN 2011 (34) AAN 2015 (35) AINI 2017 (31) CEP 2017 (36) EFSN 2010 (37) NEMOS 2014 (38)

Clinical question is as follows: in a patient with clinical signs pertaining to a PNS, are circulating TMs and/or antibodies recommended for the screening or diagnosis of any possible concomitant malignancy and its site of origin?

a in parenthesis the malignancy/ies reported to be more frequently associated to every antibody;

b neuronal surface antibodies are written in italics;

c *in parenthesis the guidelines supporting every statement*.

*TMs, tumor markers; Abs, antibodies; PNS, paraneoplastic neurologic syndrome; NR, not reported; SCLC, small cell lung cancer; NSCLC, non-small cell lung cancer*.

**Table 4:** Recommended strategy for the detection of associated malignancies in each PNS.

**Suspected syndrome**	**Information on cancer diagnosis/screening*^***a***^***
**CLASSICAL CENTRAL NERVOUS SYSTEM**	
Limbic encephalitis	Small cell lung cancer–studies include high-resolution computed tomography (CT) of the chest and PDG-PET if the CT scan is negative [EFNS 2011-pns (29)] Testicular cancer–evaluated with ultrasound, followed by CT of the pelvic region [EFNS 2011-pns (29)] Thymoma–CT of the thorax (followed by FDG-PET) or integrated FDG-PET/CT are suggested [EFSN 2011-pns (29)] Ovarian teratoma–TV ultrasound, followed by CT/MRI-pelvis/abdomen are suggested. If negative, followed by CT of the thorax [EFSN 2011-pns (29)]
Opsoclonus- myoclonus	Initial investigation in adult patients is high-resolution CT of the chest and abdomen, and gynecological examination and mammography in women. When this evaluation is negative, FDG-PET should be considered [EFSN 2011-pns (29)]
Subacute cerebellar degeneration	If small cell lung cancer is suspected, the tumor is generally demonstrated by high-resolution CT of the chest. The use of FDG-PET should be reserved for patients with onconeural antibodies when conventional imaging fails to identify a tumor. In patients without onconeural antibodies, the sensitivity and specificity of FDG-PET is poor [EFNS 2011-pns (29)] If a gynecological tumor is suspected, careful breast and pelvic examination, mammography, and pelvic CT are recommended [EFNS 2011-pns (16)] If no malignancy is revealed with this initial work-up, surgical exploration and removal of the ovaries may be warranted, particularly in postmenopausal women with Yo antibodies [EFNS 2011-pns (29)]
**CLASSICAL PERIPHERAL NERVOUS SYSTEM**	
Subacute sensory neuronopathy	When high-resolution CT of the chest is negative, FDG-PET is recommended [EFNS 2011-pns (29)]
**CLASSICAL NEUROMUSCULAR JUNCTION AND MUSCLE**	
Lambert-Eaton myasthenic syndrome (LEMS)	It is recommended that all patients are examined by high-resolution chest CT, and PDG-PET if the CT scan is negative [GISMIA 2014 (32)] If the first screening is negative, oncologic surveillance should be continued by periodic screenings (every 6 months) for at least 2 years after LEMS onset [EFNS 2011-pns (29), GISMIA 2014 (32)]
Dermatomyositis	Screen all adult patients with dermatomyositis by CT-thorax/abdomen. Women are tested also by US of the pelvic region and mammography. Male patients under 50 years old should have US of the testes. Patients over 50 years old should have a colonoscopy (good practice point) [AWMF 2016 (33), EFNS 2011-t (28)] In children, specific attention should be paid to splenomegaly or lymphadenopathy [EFNS 2011-t (28)]
**NON-CLASSICAL, PERIPHERAL NERVOUS SYSTEM, AND CENTRAL NERVOUS SYSTEM**	
Peripheral nerve hyperexcitability (PNH) syndromes	Most adults warrant a post-contrast CT mediastinum scan as up to 15% of patients have a thymoma, sometimes in the absence of MG or AChR antibodies. This is combined with a high-resolution CT of the chest, as about 10% of PNH patients will have a small cell lung cancer or adenocarcinoma. Chest CT may also help detect Hodgkin's disease [EFNS 2011-pns (29)] When the initial tumor screen is negative and malignancy is still suspected, FDG-PET is the investigation of choice [EFNS 2011-pns (29)] Monitoring for up to 4 years is indicated in those at risk of lung cancer [EFNS 2011-pns (29)]

a *in parenthesis the guidelines supporting every statement*.

Classical PNS of the central nervous system such as limbic encephalitis, autoimmune encephalitis, opsoclonus-myoclonus syndrome, and subacute cerebellar degeneration were considered by six guidelines (4, 7, 23, 29–31). The most commonly associated malignancy in adults is SCLC, though other malignancy types have been described to occur with minor frequency. Five guidelines did not consider a strategy for cancer diagnosis (4, 7, 23, 30, 31), whereas one provided recommendations on the screening of cancer in patients with classical PNS without mentioning TMs (29). Onconeural Abs positivity such as anti-Hu and anti-CV2/CRMP5 have been reported to be associated with an increased risk of SCLC occurrence whereas positive anti-Yo were associated with the risk of breast or ovarian cancer. Neuronal surface Abs anti-VGCC have been reported to be associated with an increased risk of SCLC.

As concerns classical PNS of the peripheral nervous system, one guideline referred to subacute sensory neuronopathy (29). SCLC is the malignancy most frequently associated with subacute sensory neuronopathy, but breast cancer, ovarian cancer, sarcoma, or Hodgkin's disease may occasionally occur as well. This guideline recommended a strategy for screening underlying cancer, but did not consider TMs (29). The positivity for anti-Hu is a strong predictor of the presence of cancer especially for SCLC. The positivity of anti-CV2/CRMP5 is reported to be frequently associated with SCLC, neuroendocrine tumor, and thymoma; however, the absence of antibodies cannot be considered a reliable criterion to exclude the presence of an underlying cancer.

Five guidelines considered classical PNS affecting the neuromuscular junction and muscle like Lambert-Eaton myasthenic syndrome and dermatomyositis (23, 28, 29, 32, 33). SCLC is described as the most frequently underlying tumor in patients with Lambert-Eaton myasthenic syndrome; breast, ovarian, pancreatic, stomach, colorectal, and lung cancer, as well as lymphomas have been reported in patients with dermatomyositis. Three guidelines (28, 32, 33) proposed a strategy for cancer screening which did not recommend TMs; one guideline considered NSE in SCLC patients, but underlined the low diagnostic sensitivity of the marker (32). Onconeural Abs anti-SOX are associated with an increased risk of cancer in Lambert-Eaton myasthenic syndrome, anti-P155/140 and anti TIF1y in dermatomyositis, but their absence is not sufficient to rule out the presence of a tumor (33).

Peripheral nerve hyperexcitability disorders included in non-classical PNS were considered by four guidelines (4, 7, 23, 29). Associated malignancies more frequently reported are thymoma, SCLC, lung adenocarcinoma, and Hodgkin's disease. One guideline recommended to screening for cancer, but did not mention TMs (29). Only onconeural Abs anti-amphiphysin were reported to occur more frequently in paraneoplastic cases of Stiff person syndrome (4).

Neuromyelitis optica spectrum disorders were considered by six guidelines (31, 34–38). We have classified them in the “other types” category because it is rarely associated with cancer. One guideline reported that breast carcinoma was the most frequently associated malignancy (31). Guidelines did not provide recommendations for cancer screening in patients with this type of presentation.

## Discussion

PNS clinical manifestations are often indistinguishable from other more common neurologic diseases. Therefore, in clinical practice differential diagnosis between PNS and other neurologic conditions occur more frequently than should be expected, considering the low incidence of PNS, in general hospitals in particular.

Numerous guidelines for PNS management exist, but despite the general agreement among the recommendations, their implementation in clinical practice remains poor. In fact, when a patient has symptoms pertaining to a PNS, circulating TMs tests are frequently ordered. In a survey performed in three general hospitals in Italy as part of an internal quality assurance program, TMs requests were registered in recovery charts of 10.4–21.9% of consecutive patients admitted in neurological wards. In addition, from 61.6 to 84% of requested TMs were ordered in patients discharged from neurological wards without any diagnosis of malignancy (M. Gion, T. Trenti, personal communication). This empirical use of TMs in clinical practice is probably based on the anecdotal finding in sporadic patients with PNS, of increased levels of a TM even months before the clinical appearance of the malignancy. However, TMs have poor specificity (9–11), as circulating TM levels may be increased in several benign diseases, including systemic autoimmune and inflammatory diseases also involving the nervous system. In addition, TMs have a poor diagnostic sensitivity as their circulating levels are approximately proportional to tumor bulk (9–11, 39). In patients with PNS, this conflicts with the fact that TMs are usually considered for the screening of a malignancy when the tumor is not clinically apparent nor already detected by imaging techniques. A study (40) discussing strategies to avoid overuse as the next frontier to a health care of high quality, stated that “quality refers to the degree of match between health products and services, on the one hand, and the need they are intended to meet on the other” and that “health care that meets the needs is high quality, health care that does not meet the needs, is low quality.” In light of this statement, we can say that the request of a TM for the early diagnosis of cancer simply does not meet the need, and can be classified as overuse (41, 42). The overuse of TMs has several negative effects, including the risk of underestimating the presence of a cancer in the case of a false negative TM result, or the anxiety for the patients and the unnecessary workload of healthcare professionals for the additional tests needed to confirm or exclude the presence of a malignancy in the case of a false positive TM result. Therefore, the easy availability of a laboratory test does not justify its inappropriate request (43–45).

The present study provides a summary of the existing information reported in guidelines regarding the use of TMs for the detection of a tumor in patients with PNS. This is the most comprehensive review of guidelines on the use of circulating biomarkers in PNS diagnosis, looking at 18 guidelines from 13 different medical societies/associations. The systematic review highlights the consensus between recommended practices on this issue. Classical TMs are not recommended by any of the examined guidelines for the screening or the diagnosis of a malignancy in patients with a suspected PNS. In most cases, guidelines recommend searching for a tumor by clinical signs and traditional imaging techniques (i.e., CT scan of the chest, abdomen, and pelvis or by mammogram or ultrasound of the pelvis and testes). According to some guidelines (28, 29, 31), a combination of whole body FDG-PET and CT can also be considered to detect a tumor in some specific clinical situations. For example, a recently published guideline recommended performing PET scans on patients with suspected PNS (with/without onconeural Abs) and negative conventional imaging (46).

The detection of paraneoplastic antibodies, above all onconeural Abs, should be sought in patients with suspected PNS (28, 29, 31). Onconeural Abs are useful in defining the probability that a neurologic syndrome has a paraneoplastic origin leading clinicians toward the most appropriate work up (7).

Conversely, antibodies are only partially helpful in leading to the identification of a given malignancy, with the exception of onconeural Abs which are frequently associated with some specific malignancies. Paradigmatic examples are the association of anti-Hu Abs with SCLC and anti-Yo Abs with gynecological malignancies (23, 29).

In spite of the established role of antibodies in the differential diagnosis of PNS, all guidelines are in agreement with the limited value of antibodies as a tool to screen for the underlying malignancy. Accordingly, no guideline recommends the use of antibodies to detect a tumor and classical PNS presentation should prompt investigations of occult tumors regardless of the antibody status.

A study on the diagnostic accuracy for the cancer screening of panels of PNS antibody test results was recently published (47). The authors retrospectively reviewed 384 panels sent to two reference laboratories and found that diagnostic utility was poor, with a positive predictive value of 3.6% (1/28). In addition, they found that 15 patients negative for the PNS antibody, developed a malignancy on follow-up. The findings of this pragmatic study agree with the guidelines found in our systematic revision.

The present study has some limitations. First, the search for guidelines focused on PNS was systematic between 2007 and 2017, while the search for guidelines focused on oncology was updated in 2015 using documents identified in the Guide project (9). The explanation for this apparent temporal discrepancy is that these two searches were conducted as two independent sequential literature searches. At the time of completion of the oncological guidelines broader search and successive analysis, recommendations for the use of biomarkers in PNS were not found. The second literature search therefore included key terms to more specifically identify guidelines on PNS published or updated between 2007 and 2017. Also, in this second narrower search, a very small amount of information related to PNS was identified in the retrieved documents. Moreover, a successive scrutiny of guidelines published up to July 2019 confirmed the paucity of recommendations concerning the use of TM in PNS, attesting that no new relevant information emerged to affect the conclusions reported here. The second limitation of the present study relates to the absence of a quality evaluation for the selected guidelines. However, as a remarkable degree of unanimity was observed among the recommendations from the identified documents, one can presume that the quality appraisal of the guidelines would not have a real effect on the conclusions of the present study.

Notwithstanding these limitations, this study presents a novel tool which allows for the improvement of the implementation of recommendations on the role of circulating biomarkers for the screening of underlying malignancies in patients with PNS. We did not propose novel recommendations, but we summarized and compared all the recommendations produced by the existing guidelines. Considering the barriers to physician adherence to guidelines (20), we believe that the present work of synthesis may provide a more comprehensive, transparent, evidence-based, and theoretically informed rationale. The synthesized information tool may assist practitioners and stakeholders in the diffusion, dissemination, and implementation of guideline recommendations, decreasing inappropriate use of biomarkers for cancer screening in PNS, and ultimately improve the quality of health care.

### Conclusions

A consistent agreement was found among recommendations on the use of circulating biomarkers in PNS management across multiple guidelines from various societies, reflecting the strength of the evidence. Guidelines assert that classical TMs do not have any evidence of clinical utility in this context and that they should not be ordered before the diagnosis of the malignancy is confirmed. Only after the diagnosis of a malignancy can TMs be requested for the initial work-up, treatment monitoring, and follow-up when recommended by clinical practice guidelines (9–11).

Guidelines confirm that circulating onconeural Abs remain valuable markers of neurologic autoimmune reactions and are useful in the differential diagnosis of PNS. Conversely, onconeural Abs can only be partially helpful in the identification of a tumor, being useful in defining the probability that a neurologic disorder has a paraneoplastic origin. In addition, they are not useful in the follow-up of the malignancy as they do not behave as classical markers associated to tumor bulk.

Guidelines agree on recommending that the search of the tumor has to be performed by clinical signs and traditional imaging techniques.

The information synthesized here may potentially help clinicians in choosing the appropriate diagnostic tools for malignancies, also leading to a reduction of the work-up costs.

Future efforts should focus on optimizing the implementation of the recommendations in health care settings where the inappropriate use of circulating TMs and antibodies for cancer screening and PNS is still occurring.

## Data Availability Statement

The original contributions presented in the study are included in the article/Supplementary Material, further inquiries can be directed to the corresponding author/s.

## Author Contributions

PD and MG conceived the idea. AF, MG, and CT designed the study. IC and CT performed the literature review, selected articles, and extracted data. AF and CT contributed to the quality control of data and algorithms. IC, AF, MG, and CT drafted and edited the manuscript. PD, IC, BG, MG, AF, and CT contributed to the data analysis and interpretation. All authors contributed to the article, critically revised the manuscript draft, and approved the submitted version.

## Conflict of Interest

The authors declare that the research was conducted in the absence of any commercial or financial relationships that could be construed as a potential conflict of interest.

## Abbreviations

Abs: antibodies
PNS: paraneoplastic neurologic syndromes
SCLC: small cell lung cancer
TMs: tumor markers.
