# Supplementary material for: Serum Tumor Markers in Paraneoplastic Neurologic Syndromes: A Systematic Review of Guidelines
Source: Front Neurol. 2021 Jan 18;11:607553. doi: 10.3389/fneur.2020.607553 (PMC7848074; doi:10.3389/fneur.2020.607553)
Supplement: Supplementary file 1 [file Data_Sheet_1.pdf]

Supplementary Material

Guidelines Search

Supplementary Table 1. PubMed search strategy

Supplementary Table 2. Organizations and scientific societies whose websites have been consulted to search for guidelines

Study Selection

Supplementary Table 3. Selected guidelines focused on oncology

Supplementary Table 4. Selected guidelines focused on paraneoplastic neurologic syndromes

Additional Table

Supplementary Table 5. Summary of recommendations and supplementary information concerning circulating antibodies for the characterization of paraneoplastic neurologic syndromes

Supplementary Table 1. PubMed search strategy

**Section A: PubMed search strategy for guidelines on oncology**
"guideline OR recommendation OR consensus OR consensus development conference" AND "neoplasms OR carcinoma OR cancer OR tumor".
**Section B: PubMed search strategy for guidelines on PNS**
**#1).** (((((paraneoplastic AND (neoplasms[MeSH] OR carcinoma[MeSH] OR cancer[tiab] OR neoplasm[tiab] OR neoplasms[tiab] OR tumor[tiab] OR tumour[tiab] OR tumors[tiab] OR tumours[tiab] OR carcinoma[tiab])))) OR ((paraneoplastic neurologic syndrome OR anti-NMDA Receptor encephalitis OR Autonomic neuropathy OR Brainstem encephalitis OR Cerebellar ataxia OR Cerebellar degeneration OR Chronic pseudo-obstruction OR Dermatomyositis OR Lambert-Eaton myasthenic syndrome OR Limbic encephalitis OR Morvan's syndrome OR Neuromyelitisoptica OR Neuromyotonia OR Opsoclonus-myoclonus OR Paraneoplastic Encephalomyelitis OR Paraneoplastic Myelitis OR Paraneoplastic peripheral nerve hyperexcitability OR Paraneoplastic retinopathy OR Progressive encephalomyelitis with rigidity and myoclonus OR Sensory neuronopathy OR Stiff-person syndrome OR Subacute motor neuronopathy))))
**#2).** (guideline*[ti] OR recommendation*[ti] OR consensus[ti] OR practice guideline[pt] OR guideline[pt] OR consensus development conference[pt] OR consensus development conferences as topic[MeSH] OR consensus[MeSH])
**#3).** #1. AND #2.

Supplementary Table 2. Organizations and scientific societies whose websites have been consulted to search for guidelines

Name	Acronym	Country
American Academy Neurology	AAN	USA
American Association of Immunologists	AAI	USA
American Autoimmune Related Disorder	AARD	USA
American Neurological Association	ANA	USA
Association of American Medical Colleges	AAMC	USA
Association of British Neurologists	ABN	UK
Association of University Professors of Neurology	AUPN	USA
Associazione Demenze SIN-DEM	SINDEM	Italy
Associazione Italiana di Medicina del Sonno	AIMS	Italy
Associazione Italiana di Miologia	AIM	Italy
Associazione Italiana di Neuroepidemiologia	NEP	Italy
Associazione Italiana di Neuroimmunologia	AINI	Italy
Associazione Italiana di Neuropatologia e Neurobiologia clinica	AINPeNC	Italy
Associazione Italiana per lo studio del sistema Neurovegetativo	AINV	Italy
Associazione Neurologica Italiana per la Ricerca sulle Cefalee	ANIRCEF	Italy
Associazione per lo Studio del Sistema Nervoso Periferico	ASNP	Italy
British Society for Immunology	BSI	UK
Canadian Medical Association	CMA	Canada
Canadian Neurological Society	CNS	Canada
Clinical Immunology Society	CIS	USA
Cochrane Neurosciences Field	CNF	international
European Academy of Neurology	EAN	Europe
European Federation of Immunological Societies	EFIS	Europe
European School of Neuroimmunology	ESNI	Europe
Federation of Clinical Immunology Societies	FOCIS	USA
Federazione delle Società Medico-Scientifiche Italiane	FISM	Italy
Fondazione GIMBE (Gruppo Italiano per la Medicina basata sulle Evidenze)	GIMBE	Italy
Grading of Recommendations Assessment, Development and Evaluation Working Group	GRADE	international
Guideline Central	GC	USA
Guidelines Internetional Network	GIN	international
International Society of Neuroimmunology	ISNI	international
Lega Italiana contro l'Epilessia	LICE	Italy
Lega Italiana per la lotta contro la Malattia di Parkinson, le sindromi extrapiramidali e le demenze	LIMPE	Italy
National Institute for Health and Care Excellence	NICE	UK
National Organization Rare Disorder	NORD	USA
Norwegian Neurological Association	NNA	Norway
Società dei Neurologi, Neurochirurghi e Neuroradiologi Ospedalieri	SNO	Italy
Società Italiana di Immunologia Clinica e Allergologia	SIICA	Italy
Società Italiana di Neurofisiologia Clinica	SINC	Italy

**Name**	**Acronym**	**Country**
Società Italiana di Neurologia	SIN	Italy
Società Italiana di Neuroscienze	SINS	Italy
Swedish Neurological Society / Svenska Neurologföreningen	SNS	Sweden
The Canadian Society for Immunology	CSI	Canada
Union Européenne des Médecins Spécialistes European Union of Medical Specialists	UEMS	Europe
World Federation of Neurology	WFN	international

Supplementary Table 3. Selected guidelines focused on oncology

Guideline acronym (ref)	Guideline title	Producer	Country of production	Tumor addressed
ACCC 2012 (26)	Renal cell carcinoma. Nation-wide guideline	Association of Comprehensive Cancer Centres	The Netherlands	Renal cell carcinoma
ACCP 2013 (23)	Diagnosis and management of lung cancer, 3rd ed: American College of Chest Physicians evidence-based CPG	American College of Chest Physicians	USA	Lung cancer
CCO 2014 (24)	Referral of suspected lung cancer by family physicians and other primary care providers.	Cancer Care Ontario	Canada	Lung cancer
EFNS 2011-t (28)	Screening for tumours in paraneoplastic syndromes: report of an EFNS task force	European Federation of Neurological Society	Europe	Multiple tumors
DGP-DKG 2011 (25)	Prevention, diagnosis, therapy, and follow-up of lung cancer: interdisciplinary guideline of the German Respiratory Society and the German Cancer Society	German Respiratory Society, German Cancer Society	Germany	Lung cancer
ENETS 2015 (27)	Pulmonary neuroendocrine (carcinoid) tumors: European Neuroendocrine Tumor Society expert consensus and recommendations for best practice for typical and atypical pulmonary carcinoids.	European Neuroendocrine Tumor Society	Europe	Pulmonary neuro-endocrine (carcinoid) tumors

Supplementary Table 4. Selected guidelines focused on paraneoplastic neurologic syndromes

**Guideline acronym (ref)**	**Guideline title**	**Producer**	**Country of production**	**Paraneoplastic neurologic syndromes addressed**
AAN 2011 (34)	Evidence-based guideline: clinical evaluation and treatment of transverse myelitis: report of the Therapeutics and Technology Assessment Subcommittee of the American Academy of Neurology	American Academy of Neurology	USA	Neuromyelitis optica
AAN 2015 (35)	International consensus diagnostic criteria for neuromyelitis optica spectrum disorders	American Academy of Neurology	USA	Neuromyelitis optica
ACCP 2013 (23)	Diagnosis and management of lung cancer, 3rd ed: American College of Chest Physicians evidence-based CPGs	American College of Chest Physicians	USA	Opsoclonus-myoclonus syndrome<br>Subacute cerebellar degeneration<br>Lambert-Eaton myasthenic syndrome<br>Dermatomyositis<br>Acquired neuromyotonia
AINI 2017 (31)	Diagnostics of paraneoplastic neurological syndromes	Italian Association of Neuroimmunology	Italy	Autoimmune encephalitis<br>Neuromyelitis optica
AWMF 2016 (33)	Guidelines on dermatomyositis--excerpt from the interdisciplinary S2k guidelines on myositis syndromes by the German Society of Neurology	German Society of Neurology for German Medical Society	Germany	Dermatomyositis
CEP 2017 (36)	Diagnosis and management of Neuromyelitis Optica Spectrum Disorder (NMOSD) in Iran: A consensus guideline and recommendations	consensus expert panel	Iran	Neuromyelitis optica
EFNS 2010 (37)	EFNS guidelines on diagnosis and management of neuromyelitis optica	European Federation of Neurological Society	Europe	Neuromyelitis optica
EFNS 2011-t (28)	Screening for tumours in paraneoplastic syndromes: report of an EFNS task force	European Federation of Neurological Society	Europe	Dermatomyositis
EFNS 2011-pns (29)	Paraneoplastic neurological syndromes	European Federation of Neurological Society	Europe	Limbic encephalitis<br>Opsoclonus-myoclonus syndrome<br>Subacute cerebellar degeneration<br>Subacute sensory neuronopathy<br>Lambert-Eaton myasthenic syndrome<br>Acquired neuromyotonia

Guideline acronym (ref)	Guideline title	Producer	Country of production	Paraneoplastic neurologic syndromes addressed
EFNS 2012-cns (4)	Central nervous system neuronal surface antibody associated syndromes: review and guidelines for recognition	European Federation of Neurological Society	Europe	Limbic encephalitis<br>Autoimmune encephalitis<br>Subacute cerebellar degeneration<br>Opsoclonus-myoclonus syndrome<br>Stiff person syndrome
EFNS 2012-d (30)	EFNS-ENS Guidelines on the diagnosis and management of disorders associated with dementia	European Federation of Neurological Society	Europe	Limbic encephalitis
GISMIA 2014 (32)	Italian recommendations for Lambert-Eaton myasthenic syndrome (LEMS) management	Italian myasthenic syndrome working group	Italy	Lambert-Eaton myasthenic syndrome
PNSE 2004 (7)	Recommended diagnostic criteria for paraneoplastic neurological syndromes	Paraneoplastic Neurological Syndrome Euronetwork	Europe	Limbic encephalitis<br>Subacute cerebellar degeneration<br>Stiff person syndrome
NEMOS 2014 (38)	Update on the diagnosis and treatment of neuromyelitis optica: recommendations of the Neuromyelitis Optica Study Group (NEMOS)	Neuromyelitis Optica Study Group	Germany	Neuromyelitis optica

Supplementary Table 5. Summary of recommendations and supplementary information concerning circulating antibodies for the characterization of PNS

**Suspected syndrome**	**Antibodies to be considered for the characterization of the PNS**[a,b]	**Summary of recommendations and supplementary information concerning the use of antibodies for the characterization of the PNS**[a]	**Guidelines that consider the specific PNS**
Classical Central Nervous System			
Limbic encephalitis (LE)	anti-Hu (ACCP 2013, EFNS 2011-pns, PNSE 2004)<br>anti-Ma2 (within/out anti-Ma1) (EFNS 2011-pns, PNSE 2004)<br>anti-CV2/ CRMP5 (EFNS 2011-pns, PNSE 2004)<br>anti-amphiphysin (EFNS 2011-pns)<br>*anti-VGKC complex (anti-LGI1, anti-CASPR2)* (EFNS 2011-pns, EFNS 2012-cns)<br>*anti-GluR1* (EFNS 2011-pns)<br>*anti-AMPA-R* (EFNS 2011-pns, EFNS 2012-cns)<br>*anti-GAD* (EFNS 2011-pns, EFNS 2012-cns)<br>*anti-GABAb-R* (EFNS 2012-cns)<br>*anti-mGluR5* (EFNS 2012-cns)	Onconeural antibodies that target intracellular antigens (Hu, Ma2, CV2/CRMP5, amphiphysin, GAD) are tightly associated with the presence of an underlying tumour; antibodies against surface receptors (LGI1, CASPR2, AMPA-R, NMDA-R, GluR1, GABAb-R, mGluR5) are present in patients with limbic encephalitis that may or may not be paraneoplastic. (EFNS 2011-pns)<br>Antineuronal antibodies should be measured both in serum and in cerebrospinal fluid. (EFNS 2011-pns)	EFSN 2011-pns (29)<br>EFNS 2012-cns (4)<br>EFNS 2012-d (30)<br>PNSE 2004 (7)
Autoimmune Encephalitis (AE)	*anti-NMDA-R* (AINI 2017, EFNS 2011-pns, EFNS 2012-cns)<br>*anti-VGKC complex (LGI1, CASPR2)* (AINI 2017, EFNS 2012-cns, EFNS 2011-pns)<br>*anti-AMPA-R* (AINI 2017)<br>*anti-D2R* (AINI 2017)<br>*anti-DPPX* (AINI 2017)<br>*anti-GABAa-R* (AINI 2017)<br>*anti-GABAb-R* (AINI 2017)<br>*anti-Gly-R* (AINI 2017)<br>*anti-IgLON5* (AINI 2017)<br>*anti-mGluR5* (AINI 2017)<br>*anti-Neurexin-3α* (AINI 2017)<br>Morvan's syndrome<br>*anti-VGKC complex* (EFNS 2011-pns)	Both serum and cerebrospinal fluid samples should be tested in routine diagnostics, when NS-Ab-associated AE is suspected. (AINI 2017)<br>Patients with serum positivity and cerebrospinal fluid negativity, or with cerebrospinal fluid sample unavailable, should be considered with high caution. (AINI 2017)	AINI 2017 (31)<br>EFNS 2012-cns (4)
Opsoclonus - Myoclonus (OMS)	anti-Hu (ACCP 2013, EFNS 2011-pns)<br>anti-amphiphysin (EFNS 2011-pns)<br>anti-Ri (ACCP 2013, EFNS 2011-pns)<br>anti-Ma2 (EFNS 2011-pns)	Most infant and adult patients do not harbour a clearly defined onconeural antibody. (EFSN 2011-pns)	ACCP 2013 (23)<br>EFSN 2011-pns (29)<br>EFNS 2012-cns (4)

**Suspected syndrome**	**Antibodies to be considered for the characterization of the PNS[a,b]**	**Summary of recommendations and supplementary information concerning the use of antibodies for the characterization of the PNS[a]**	**Guidelines that consider the specific PNS**
Subacute Paraneoplastic Cerebellar Degeneration (PCD)	anti-Yo (ACCP 2013, EFNS 2011-pns, PNSE 2004)<br>anti-CV2/CRMP5 (EFNS 2011-pns, PNSE 2004)<br>anti-Hu (ACCP 2013, EFNS 2011-pns, PNSE 2004)<br>anti-Ri (EFNS 2011-pns, PNSE 2004)<br>Some association with:<br>anti-amphiphysin (EFNS 2011-pns)<br>anti-Ma2 (EFNS 2011-pns)<br>anti-Tr (EFNS 2011-pns, PNSE 2004)<br>anti-Zic4 (EFNS 2011-pns, PNSE 2004)<br>*anti-VGCC* (EFNS 2011-pns, EFNS 2012-cns, PNSE 2004)<br>*anti-mGluR1* (EFNS 2011-pns, EFNS 2012-cns, PNSE 2004)<br>*anti-GAD* (EFNS 2012-cns)	The absence of onconeural antibodies cannot rule out the diagnosis of PCD, as only 50% of patients with PCD harbour such antibodies. (EFNS 2011-pns)<br>Anti-GAD cerebellar degeneration are non paraneoplastic. (EFNS 2012-cns)	ACCP 2013 (23)<br>EFSN 2011-pns (29)<br>EFNS 2012-cns (4)<br>PNSE 2004 (7)
Classica Peripheral Nervous System			
Subacute Sensory Neuronopathy (SSN)	anti-Hu (ACCP 2013, EFNS 2011-pns, PNSE 2004)<br>anti-CV2/CRMP5 (EFNS 2011-pns, PNSE 2004)	The absence of anti-Hu does not exclude the presence of an underlying cancer. (EFNS 2011-pns)<br>Anti-CV2/CRMP5 neuropathy is usually sensory or sensory-motor, in which upper limbs are less frequently involved, but often associated with cerebellar ataxia. (EFNS 2011-pns)	EFSN 2011-pns (29)
Classical Neuromuscolar Junction and Muscle			
Lambert-Eaton Myasthenic Syndrome (LEMS)	*anti-VGCC (P/Q type, N type)* (ACCP 2013, EFNS 2011-pns, GISMIA 2014)<br>anti-SOX (EFNS 2011-pns, GISMIA 2014)	More than 75-90% LEMS have antibodies against VGCC type P/Q. (ACCP 2013, EFNS 2011-pns, GISMIA 2014)<br>The absence of these antibodies does not exclude a diagnosis of LEMS, though it reduces the probability of association with SCLC. (GISMIA 2014)<br>Non-tumour LEMS is often associated with HLA-B8, DR3 haplotype and to other autoimmune diseases (es. rheumatoid arthritis, pernicious anaemia, thyroid disorders, Sjogren's syndrome). (GISMIA 2014)	ACCP 2013 (23)<br>EFSN 2011-pns (29)<br>GISMIA 2014 (32)
Dermatomyositis (DM)	No particular paraneoplastic antibodies have been described (ACCP 2013, EFNS 2011-t)<br>anti-Mi2 (AWMF 2016)	Anti-Mi2 sometimes occur in paraneoplastic DM, high titers can also be found in juvenile DM. (AWMF 2016)<br>Tests for some autoantibodies, which are not yet part of the routine diagnostic workup, should be predominantly used in a scientific context. Especially anti-P155/140 or anti-TIF1y-Ab, anti-Mup44-Ab and anti-HMG CoA reductase which are frequently associated with paraneoplastic DM. (AWMF 2016)	ACCP 2013 (23)<br>AWMF 2016 (33)<br>EFNS 2011-t (28)

Suspected syndrome	Antibodies to be considered for the characterization of the PNS[a,b]	Summary of recommendations and supplementary information concerning the use of antibodies for the characterization of the PNS[a]	Guidelines that consider the specific PNS
Non-Classical, Peripheral Nervous System and Central Nervous System			
Peripheral Nerve Hyperexcitability (PNH) syndromes (Stiff person, Neuromyotonia, Progressive Encephalomyelitis with rigidity and myoclonus-PERM)	Stiff Person syndrome:<br>*anti-GAD* (EFNS 2012-cns)<br>anti-amphiphysin (EFNS 2012-cns, PNSE 2004)<br>Neuromyotonia:<br>*anti-VGKC* (ACCP 2013, EFNS 2011-pns)<br>anti-Hu (EFNS 2011-pns)<br>PERM:<br>*anti-GAD* (EFNS 2012-cns)<br>*anti-GlyR* (EFNS 2012-cns)	There is no antibody that indicates whether PNH is paraneoplastic. (EFNS 2011-pns)<br>Anti-amphiphysin are more frequent in paraneoplastic cases of Stiff Persons. (EFNS 2012-cns)	ACCP 2013 (23)<br>EFNS 2011-pns (29)<br>EFNS 2012-cns (4)<br>PNSE 2004 (7)
Other			
Neuromyelitis optica spectrum disorders (NMOSD)	*anti-AQP4* (AAN 2011, AAN 2015, AINI 2017, CEP 2017, EFNS 2010, NEMOS 2014)<br>*anti-MOG* (AAN 2015, AINI 2017, NEMOS 2014)	Testing of anti-AQP4 is essential and is the most important test in the diagnostic work-up of suspected NMOSD, and helps to distinguish NMO from other encephalomyelitis/myelitis. (AAN 2011, AAN 2015, AINI 2017, CEP 2017, EFNS 2010, NEMOS 2014)<br>A minority of patients with clinical characteristics of NMO, AQP4-IgG-seronegative, have detectable serum anti-MOG (AAN 2015, NEMOS 2014), however, the exact diagnostic and therapeutic relevance of this finding is currently investigated. (NEMOS 2014)<br>Cerebrospinal fluid testing of AQP4-IgG might be considered in selected seronegative cases with additional confounding serum autoantibodies that may lead to uninterpretable or false-positive assay results. (AAN 2015, AINI 2017, EFNS 2010)<br>Occasionally, patients without detectable serum AQP4-IgG are later found to be seropositive. Therefore, retesting should be considered in seronegative patients who relapse. (AAN 2015, NEMOS 2014)<br>Standard sample is serum. (AINI 2017, EFNS 2010)<br>Routine cerebrospinal fluid testing of anti-AQP4 IgG-seronegative patients is not recommended. (AAN 2015, CEP-2017, NEMOS 2014)	AAN 2011 (34)<br>AAN 2015 (35)<br>AINI 2017 (31)<br>CEP 2017 (36)<br>EFSN 2010 (37)<br>NEMOS 2014 (38)

a: In parenthesis the guidelines supporting every statement; *b*: Neuronal surface antibodies are written in italics
PNS= Paraneoplastic Neurologic Syndromes
